# Systemic inflammation in early neonatal mice induces transient and lasting neurodegenerative effects

**DOI:** 10.1186/s12974-015-0299-3

**Published:** 2015-04-29

**Authors:** Filipa L Cardoso, Jasmin Herz, Adelaide Fernandes, João Rocha, Bruno Sepodes, Maria A Brito, Dorian B McGavern, Dora Brites

**Affiliations:** Research Institute for Medicines (iMed.ULisboa), Faculdade de Farmácia, Universidade de Lisboa, Avenida Professor Gama Pinto, 1649-003 Lisbon, Portugal; National Institute of Neurological Disorders and Stroke, National Institutes of Health, 10 Center Drive, Bethesda, MD 20892-1430 USA; Department of Biochemistry and Human Biology, Faculdade de Farmácia, Universidade de Lisboa, Avenida Professor Gama Pinto, 1649-003 Lisbon, Portugal

**Keywords:** Astrocytes, Microglia, Myelination, Neurons, Autotaxin, HMGB1, Neurodevelopment, LPS

## Abstract

**Background:**

The inflammatory mediator lipopolysaccharide (LPS) has been shown to induce acute gliosis in neonatal mice. However, the progressive effects on the murine neurodevelopmental program over the week that follows systemic inflammation are not known. Thus, we investigated the effects of repeated LPS administration in the first postnatal week in mice, a condition mimicking sepsis in late preterm infants, on the developing central nervous system (CNS).

**Methods:**

Systemic inflammation was induced by daily intraperitoneal administration (i.p.) of LPS (6 mg/kg) in newborn mice from postnatal day (PND) 4 to PND6. The effects on neurodevelopment were examined by staining the white matter and neurons with Luxol Fast Blue and Cresyl Violet, respectively. The inflammatory response was assessed by quantifying the expression/activity of matrix metalloproteinases (MMP), toll-like receptor (TLR)-4, high mobility group box (HMGB)-1, and autotaxin (ATX). In addition, B6 CX3CR1^gfp/+^ mice combined with cryo-immunofluorescence were used to determine the acute, delayed, and lasting effects on myelination, microglia, and astrocytes.

**Results:**

LPS administration led to acute body and brain weight loss as well as overt structural changes in the brain such as cerebellar hypoplasia, neuronal loss/shrinkage, and delayed myelination. The impaired myelination was associated with alterations in the proliferation and differentiation of NG2 progenitor cells early after LPS administration, rather than with excessive phagocytosis by CNS myeloid cells. In addition to disruptions in brain architecture, a robust inflammatory response to LPS was observed. Quantification of inflammatory biomarkers revealed decreased expression of ATX with concurrent increases in HMGB1, TLR-4, and MMP-9 expression levels. Acute astrogliosis (GFAP^+^ cells) in the brain parenchyma and at the microvasculature interface together with parenchymal microgliosis (CX3CR1^+^ cells) were also observed. These changes preceded the migration/proliferation of CX3CR1^+^ cells around the vessels at later time points and the subsequent loss of GFAP^+^ astrocytes.

**Conclusion:**

Collectively, our study has uncovered a complex innate inflammatory reaction and associated structural changes in the brains of neonatal mice challenged peripherally with LPS. These findings may explain some of the neurobehavioral abnormalities that develop following neonatal sepsis.

## Background

Sepsis is a common severe inflammatory response to an infection that may afflict people at any age. Patients have a high risk of morbid complications and death. Fatality rates range from 10% to 20% in sepsis, to 20% to 50% in severe sepsis, and 40% to 80% in septic shock [1]. Neonatal septicemia (first 28 days of life) is the third leading cause of death in developed and developing countries, and a gestational age of less than 32 weeks is considered to be a risk factor for this disease [2-4]. Induction of lipopolysaccharide (LPS)-induced sepsis during the first postnatal week in mice [5,6] reproduces many of the complications observed in late premature human babies with septicemia [7]. Systemic administration of LPS, an endotoxin of gram-negative bacteria, is widely used to induce a neuroinflammatory response associated with short-term ‘sickness’ behavior [8] in adult [9] and newborn animals [10] as well as during gestation [11]. In these models, weight loss is a commonly observed sign of illness [12-14] and is one of the consequences of sepsis [15,16]. Across species, sepsis survivors frequently experience white matter damage [17], cerebral palsy [18], as well as cognitive and affective disorders [19]. A single systemic LPS injection is intended to reproduce the acute systemic LPS-mediated inflammation [8], whereas a repetitive challenge is used to model a chronic condition [20,21].

Although LPS entrance into the brain is low [22], acute systemic inflammation is known to induce transient expression of proinflammatory mediators and microglia activation but only to a mild extent and without neuronal death [23]. However, in contrast with a single LPS application, repeated systemic challenge of mice was shown to sustain the microglial inflammatory phenotype, trigger the loss of neurons [24], and produce changes in cerebral vasculature that include upregulation of the major histocompatibility complex (MHC) class I and II [20].

How peripheral LPS induces its effects on brain is still unclear, but mechanisms may involve alterations in the blood-brain barrier (BBB) permeability and function, stimulation of LPS receptors located outside the BBB [22,25], or activation of brain microvascular endothelial cells (BMECs) through the induction of downstream signaling pathways [26]. BMECs physically separate the brain from the blood, forming the basis of the BBB. These cells express the toll-like receptor (TLR)-4, whose activation by LPS leads to the synthesis and release of pro-inflammatory cytokines [27,28]. In addition, LPS triggers the release of activated matrix metalloproteinase (MMP)-9 and MMP-2 as well as granulocyte-macrophage colony-stimulating factor, which may contribute to the infiltration of monocytes and to BBB breakdown [29,30]. BMECs are part of the neurovascular unit that also includes the basement membrane, astrocytic endfeet, pericytes, microglia, neurons, and even oligodendrocytes [31]. The direct response of these cell types to inflammatory stimuli or the released pro-inflammatory signals may exacerbate the damaging effects on BMECs [26,32].

Sensitive time windows for LPS-induced alterations in neurodevelopment result from the fact that neuronal migration, gliogenesis, and myelinogenesis occur at a late gestational age and predominate in the first 2 weeks of postnatal life [33]. Microvessel ensheathment by astrocyte endfeet takes place during the first postnatal week meaning that some barrier properties should be acquired after birth, at least in rodents [34]. In addition, LPS triggers astrocytic production of pro-inflammatory cytokines, particularly in immature cells [35], directly influencing neurodegeneration [36]. LPS also induces brain-resident immune cells like microglia to release pro-inflammatory cytokines and other inflammatory mediators such as the high-mobility group box 1 (HMGB1) [37] and autotaxin (ATX) [38] that mediate changes in neuronal network activity and apoptosis [39]. Though microglia progenitors colonize the mouse brain early in embryogenesis, the main transition from amoeboid into a ramified shape occurs during the second week after birth, along with increased microglial numbers and the maturation of neurons [28,40]. The morphological features of microglia and their colonization of the mouse brain are similar in humans [41].

A robust inflammatory response to LPS is mounted during the acute phase (first hours), which to some degree is counterbalanced by an anti-inflammatory response during the later stages [42]. Despite induction of an anti-inflammatory program, studies have shown that repeated injection of LPS (prolonged sepsis) can potentiate proinflammatory cytokine levels in the brain [43]. It is therefore important to understand the pathogenesis of sepsis and its sequelae when proinflammatory cytokine levels are sustained in the developing neonatal brain for days in order to develop novel ways to improve survival and preventing adverse outcomes. Indeed, most of the studies have been performed in young or aged mice [20,44-46] instead of the neonatal period [47]. Thus, we set out to investigate the effects of repetitive LPS injections during the first postnatal week on murine development. We found that sustained systemic inflammation interferes with central nervous system (CNS) maturation by causing neuronal atrophy, a delay in myelination, and acute reactive gliosis.

## Materials and methods

### Animals

Pregnant CD1 *wild-type* (WT) mice at embryonic day (E)16 were purchased from Harlan Ibérica (Spain) laboratories and gave birth in the animal facility of the Faculdade de Farmácia, Universidade de Lisboa. To better explore microglia activation, we used heterozygous C57BL/6 (B6) CX3CR1^gfp/+^ mice. These mice were generated by crossing B6 WT with B6 CX3CR1^gfp/gfp^ mice that were purchased from The Jackson Laboratory (Bar Harbor, ME, USA) and maintained in a closed breeding facility at The National Institutes of Health (NIH), Bethesda. The insertion of the green fluorescent protein (GFP) allows the tracking of CX3CR1^+^ cells, which is important to visualize microglia dynamic changes [48,49]. Homozygous B6 CX3CR1^gfp/gfp^ mice are CX3CR1 deficient and do not respond to fractalkine. On the other hand, both B6 WT and CX3CR1^gfp/+^ mice respond similarly to LPS [50].

Mice were housed with a 12-h light/dark cycle and were provided with *ad libitum* access to a standard laboratory chow diet and drinking water. This study was carried out in strict accordance with the recommendations of European Convention for the Protection of Vertebrate Animals Used for Experimental and other Scientific Purposes (Council Directive 86/609/EEC), as well as with those in the Guide for the Care and Use of Laboratory Animals at the NIH. The animal study protocol was approved by the NINDS Animal Care and Use Committee (Assurance Number: A4149-01). All experimental procedures were performed under anesthesia, conducted in a manner to minimize animal suffering, and all efforts were made to use the minimum number of animals.

### Drug administration

**Figure 1:**
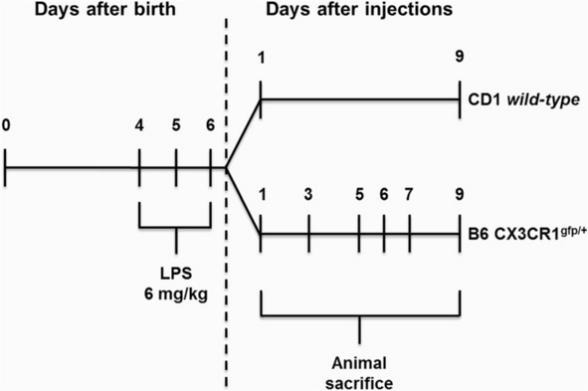
Schematic representation of the early induction of systemic inflammation. Offspring of both genders were randomly divided into two groups and treated with three intraperitoneal injections of either saline solution or lipopolysaccharide (LPS) at days 4, 5, and 6 after birth to induce systemic inflammation. CD1 wild-type mice were sacrificed at 1 and 9 days after LPS injections, and C57BL/6 (B6) CX3CR1^gfp/+^ mice were sacrificed at days 1/3/5/6/7/9 following LPS administration.

The day of birth was defined as PND1. For each strain, offspring of both genders were randomly divided into two groups and were treated from PND4 to PND6 with daily i.p. injections of either endotoxin-free saline [control (W/O LPS); *n* ≥ 4 per analysis] or of LPS [6 mg/kg, *Escherichia coli* 055:B5; Calbiochem (Merck, Darmstadt, Germany); *n* ≥ 4 per analysis] to induce systemic inflammation [51,52]. CD1 WT mice were sacrificed 1 day after the final LPS administration (LPS1) and at LPS9 to evaluate acute and lasting effects, respectively. B6 CX3CR1^gfp/+^ mice were sacrificed at LPS1/3/5/6/7/9 not only to determine the acute and lasting effects but also the delayed effects. Injection and sampling regimens are depicted in Figure 1.

### Tissue process

For paraffin-histological analysis, CD1 WT mice were perfused *via* the ascending aorta with 4% paraformaldehyde (PFA) in PBS. Brains were post-fixed in the indicated fixative for at least 24 h. Brain tissue was processed for paraffin and cut into 6-μm sagittal sections. For gelatin zymography and Western blot analysis, the same animals were perfused *via* the ascending aorta with phosphate buffer saline solution, pH 7.4 (PBS). Brains were quickly removed, snap-frozen, and cryopreserved at −80°C for at least 24 h. Protein extracts were obtained by lysing the brain tissue with radioimmunoprecipitation assay (RIPA) buffer (Tris Buffer 1 M pH 8.0, EDTA 0.5 M pH 8.0, NaCl 5 M, 10% NP-40, 50% glycerol, 10% SDS) [53]. For cryo-histological analysis, B6 CX3CR1^gfp/+^ animals were perfused with 4% PFA in PBS. Brains were post-fixed in the same fixative for 24 h, followed by 15% and 30% sucrose solutions, each for at least 16 h. Brain tissue was embedded in TFM^TM^ Tissue Freezing Medium (TBS® Triangle Biomedical Sciences, Durham, NC, USA), frozen at −80°C and cut with a cryomicrotome into 25-μm sagittal sections.

### Staining for Luxol Fast Blue and Cresyl Violet

Paraffin sections were stained with Luxol Fast Blue (VWR, Radnor, PA, USA) for oligodendrocyte myelin followed by with Cresyl Violet (Sigma, St. Louis, MO, USA) for neuronal Nissl bodies’ assessment. Sections were rehydrated in xylene for 20 min and decreasing concentrations of ethyl alcohol for 10 min each. Sections were then incubated with a 0.1% Luxol Fast Blue solution in 70% ethyl alcohol overnight at 56°C. After rinsing with 70% ethyl alcohol followed by distilled water to remove excess stain, the tissue was differentiated in a 0.5% lithium carbonate solution. Slides were then rinsed in distilled water followed by 2% acetic acid solution for 5 min. Next, tissue was counterstained with a 1% Cresyl Violet solution for 10 min. After rinsing in distilled water, sections were differentiated in 37.5% acetic acid and rinsed in distilled water. Slides were mounted with Fluoromount-G (Southern Biotech, Birmingham, AL, USA) and visualized using a DFC 490 camera (Leica, Wetzlar, Germany) adapted to an Axioskop bright field microscope (Zeiss, Oberkochen, Germany). Width of cerebellar layers, number of neurons per field, and the area of their soma were analyzed using ImageJ software (NIH, Bethesda, MD, USA). Intensity of staining of the external germinal layer at LPS1 and of the white matter layer at LPS9 was analyzed using the same software and was expressed per square micrometers.

### Gelatin zymography

Determination of MMP-9 and MMP-2 was evaluated as previously described [29] with minor alterations. In short, 40 μg of protein from tissue extracts were analyzed by SDS-PAGE zymography in 0.1% gelatin/10% acrylamide gels under non-reducing conditions. After electrophoresis, gels were washed for 1 h with 2.5% Triton X-100 (in 50 mM Tris pH 7.4, 5 mM CaCl_2_, 1 mM ZnCl_2_) to remove SDS and renature the MMPs species in the gel. Gels were then incubated in developing buffer (50 mM Tris pH 7.4, 5 mM CaCl_2_, 1 mM ZnCl_2_) for 72 h at 37°C to induce gelatin lysis. For enzyme activity analysis, gels were stained with 0.5% Coomassie Brilliant Blue R-250 (Bio-Rad, Hercules, CA, USA) for 3 h at room temperature (RT) and distained in 30% ethanol/10% acetic acid/H_2_O. Gelatinase activity, detected as a white band on a blue background, was quantified by computerized image analysis using Quantity One 1-D Analysis Software (Bio-Rad, Hercules, CA, USA).

### Western blot

Western blot analysis was performed as previously described [53] with minor alterations. Briefly, 100 μg of protein from tissue extracts were separated on a 12% SDS-PAGE gel. Following electrophoretic transfer onto a nitrocellulose membrane and blocking with 5% milk solution, the blots were incubated with primary antibody overnight at 4°C [rabbit anti-TLR4 (Santa Cruz, Dallas, TX, USA, #sc-10741; 1:500), mouse anti-HMGB1 (BioLegend, San Diego, CA, USA, #651402; 1:500), rabbit anti-ATX (Millipore, Billerica, MA, USA, #ABT28; 1:500), or mouse anti-β-actin (Sigma, USA, #A5441; 1:5,000)] and with horseradish peroxidase-labeled secondary antibody [anti-mouse or anti-rabbit (Santa Cruz, USA, #sc-2005 and #sc-2004, respectively; 1:5,000)] for 1 h at RT. Protein bands were detected by LumiGLO® (Cell Signaling, Danvers, MA, USA) and visualized by chemiluminescence with ChemiDoc (Bio-Rad, Hercules, CA, USA). Expression was quantified by computerized image analysis using Quantity One 1-D Analysis Software (Bio-Rad, Hercules, CA, USA).

### Cryo-immunofluorescence

Frozen sections were used to analyze the expression of the fractalkine receptor, CX3CR1, as well as NG2^+^ glia, GFAP, myelin basic protein (MBP), and cluster of differentiation (CD)31 in B6 CX3CR1^gfp/+^ mice. Sections were fixed for 15 min with 1% PFA in PBS. Sections were then treated with an Avidin/Biotin Blocking Kit (Vector Laboratories, Burlingame, CA, USA, #SP-2001) per the manufacturer’s instructions followed by 20-min treatment with Background Buster (INNOVEX Biosciences, Richmond, CA, USA, #NB306). Tissue was incubated 1 h at RT with primary antibodies: rabbit anti-NG2 (Millipore, USA, #AB5320; 1:100), rabbit anti-GFAP (DAKO, Glostrup, Denmark #Z0334; 1:200), rat anti-MBP (Millipore, Billerica, MA, USA, #MAB386; 1:100), or Armenian hamster anti-CD31, clone 2H8 (Chemicon, Temecula, CA, USA, #MAB1398Z; 1:200). Following the incubation with primary antibodies, sections were washed and incubated for 1 h at RT with secondary antibodies (all from Jackson ImmunoResearch Laboratories, West Grove, PA, USA) donkey Alexa Fluor 647 anti-rabbit IgG (H&L) (#711-605-152; 1:200), donkey Alexa Fluor 647 F(ab) anti-rat IgG (H&L) (#712-606-150; 1:200), or goat Rhod-X anti-Armenian hamster IgG (H&L) (#127-295-160; 1:200). CD31 staining was amplified with donkey Rhod-X F(ab) anti-goat IgG (H&L) (#705-296-147; 1:200) for 1 h at RT. All working stocks of primary and secondary reagents were diluted in PBS containing 2% fetal bovine serum (FBS) + 0.5% Triton X-100. Nuclei were counterstained with DAPI dye, and sections were mounted with IMMU-MOUNT (Thermo-Scientific, Waltham, MA, USA, #9990402). Between incubations, sections were washed three times with PBS. Apoptosis was detected in frozen sections with the ApopTag® Red *In Situ* Apoptosis Detection Kit (Chemicon, Temecula, CA, USA, #S7165), which specifically detects DNA cleavage and chromatin condensation associated with apoptosis, in accordance with the manufacturer’s instructions. Images were captured from stained frozen sections using an Olympus FV1200 confocal microscope equipped with 20× and 40× objectives. Images were collected using sequential scanning with the 405-, 488-, 559-, and 635-nm laser lines to produce four color overlays. Cerebellar area was measured in tiles of DAPI-counterstained brain sections using ImageJ (NIH, Besthesda, MD, USA). Area fraction and colocalization of the staining per field of each protein was quantified by computerized image analysis using ImageJ (NIH, Besthesda, MD, USA).

### Sholl analysis of CX3CR1^+^ cells

To quantify morphological changes of the CX3CR1^+^ cells, consecutive Z-stack images were converted to a maximum intensity projection image by ImageJ software (NIH Besthesda, MD, USA). Using the Sholl analysis plugin, concentric circles were created centered on the soma, beginning at 5.5-μm radii and increasing 2 μm with every circle. We determined the number of intersections made by microglia branching processes with each successive increasing circle, the maximum number of intersections for the cell (Nm), as well as the critical value at which Nm occurred and the maximum length at which a branch intersection was observed [54].

### Statistical analysis

Results are expressed as means ± SEM from, at least, four independent animals in each treatment group. Significant differences between groups were determined by the two-tailed *t*-test performed on the basis of equal and unequal variance as appropriate. Comparison of more than two groups was done by ANOVA using GraphPad Prism® 5.0 (GraphPad Software, San Diego, CA, USA). Statistical significance was considered when *P* values were lower than 0.05.

## Results

### LPS administration in the early neonatal period triggers acute weight loss and cerebellar hypoplasia

**Figure 2:**
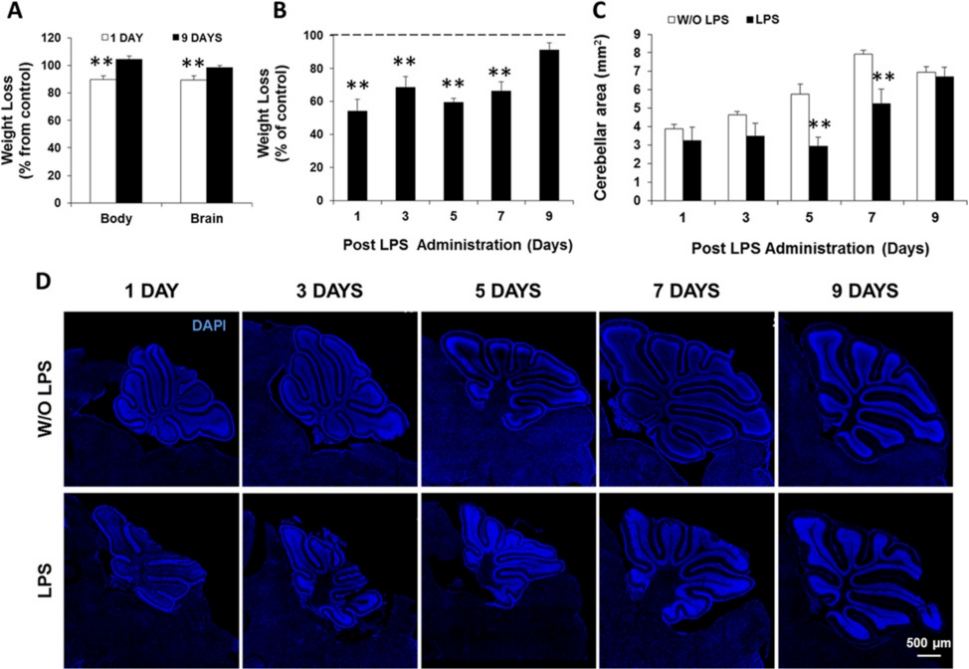
Early lipopolysaccharide (LPS) administration causes transient body and brain weight loss and decreases the cerebellar area. **(A)** Body and brain of CD1 *wild-type* mice were weighed at 1 and 9 days post-LPS administration. **(B)** Body weight of C57BL/6 (B6) CX3CR1^gfp/+^ mice was assessed at days 1/3/5/7/9 after LPS injection. **(C)** Cerebellar area was measured in tiled confocal images of brain sections from B6 CX3CR1^gfp/+^ mice at days 1/3/5/7/9 post-LPS (representative images with DAPI in **(D)**. Results are mean ± SEM from at least four animals. **P < 0.01 *vs*. without (W/O) LPS.

Administration of LPS is known to induce a sickness behavior in adult mice, including weight loss [55,56]. To assess the impact of LPS exposure in the first postnatal week, we initially assessed body and brain weight oscillations, which were acutely decreased by LPS (Figure 2A). Body weight loss was sustained up to LPS7 (*P* < 0.01) but eventually recovered by LPS9 (Figure 2B). We further explored acute brain weight loss by measuring the sagittal cerebellar area. This revealed a disruption in its development evidenced by an approximately twofold reduction at LPS5 that was still evident at LPS7 (*P* < 0.01). No difference from the control group was observed at LPS9 (Figure 2C,D).

### Induction of neuronal atrophy in newborn mice by systemic inflammation is more marked at LPS1 than at LPS9

**Figure 3:**
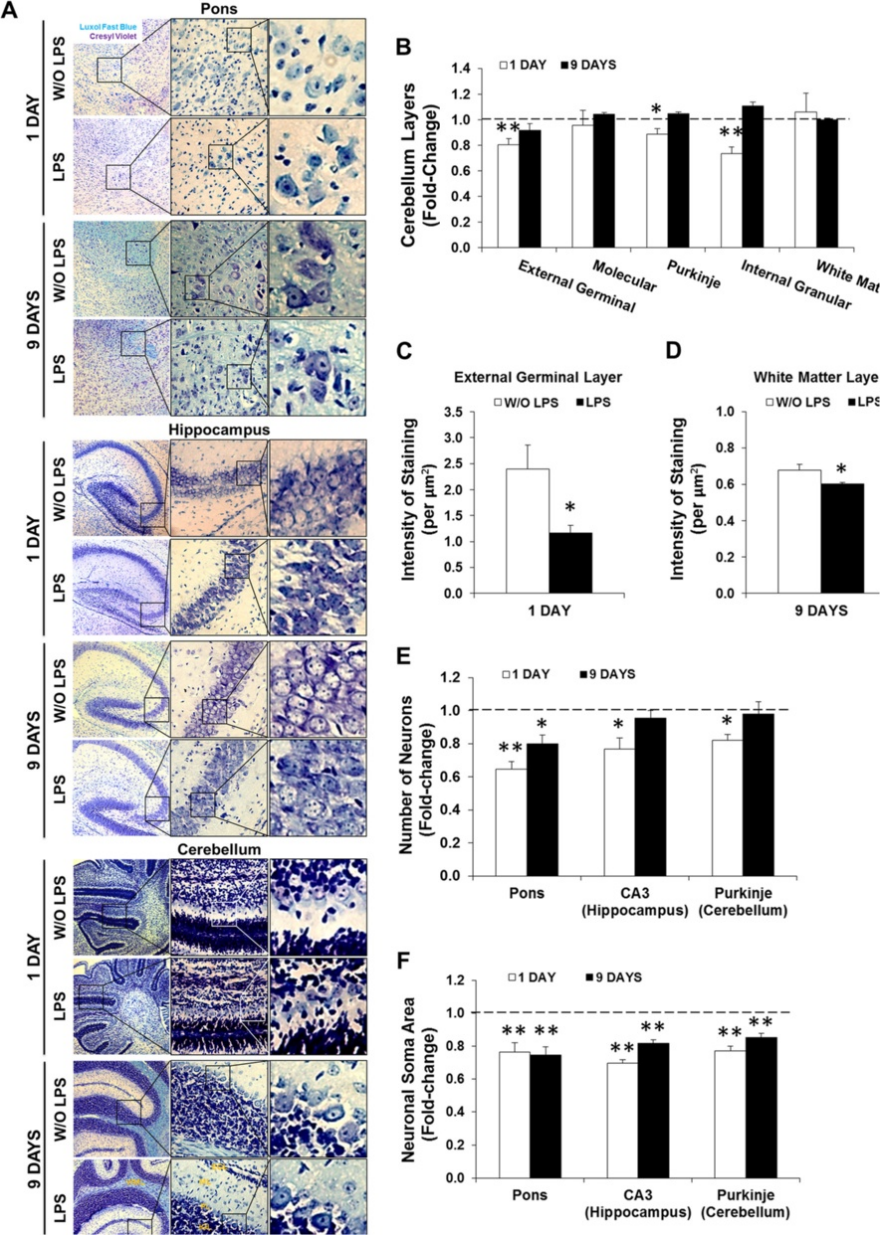
Lipopolysaccharide (LPS) administration triggers transient shrinkage of cerebellar layers, acute neuronal loss, and sustained atrophy. Paraffin sections from CD1 *wild-type* mice at 1 and 9 days post-LPS administration were stained with Luxol Fast Blue (myelin, blue) followed by Cresyl Violet (Nissl bodies, purple). **(A)** Representative images are shown for each condition in the pons, hippocampus, and cerebellum. **(B)** The widths of each cerebellar layer [external germinal layer (EGL), molecular layer (ML), Purkinje layer, internal granular layer (IGL), white matter layer (WML)] were measured. **(C,D)** Intensity of Cresyl Violet staining in the EGL and of Luxol Fast Blue in WML were quantified per square micrometer at 1 and 9 days after LPS administration, respectively. **(E,F)** The number of neurons per field and the area of neuronal cell body (soma) was quantified throughout the pons, in the CA3 hippocampal region, and in the cerebellar Purkinje layer (PL). All determinations were done using ImageJ software (NIH, USA). Results are mean ± SEM from at least five animals. *P < 0.05 *vs*. without (W/O) LPS.

Given the observed decrease in the cerebellar area, we next explored the width of each layer: the external germinal layer (with proliferating neuroepithelial cells), the molecular layer (containing the axons of granule cells and dendrites of Purkinje cells), the Purkinje neuronal layer, the granular layer (with small neurons called granule cells), and the white matter layer (with myelin fibers) (representative images of cerebellum are shown in Figure 3A). There was a significant shrinkage of the neuron-containing layers at 24 h after the last LPS injection (*P* < 0.05 for the Purkinje layer; *P* < 0.01 for the external germinal layer and granular layer) but not at LPS9 (Figure 3B). In agreement, the density of neurons based on the intensity of staining per square micrometer of cells in the external germinal layer was also markedly reduced at LPS1 (approximately twofold, *P* < 0.05) (Figure 3C). Lastly, a negative impact on the density of Luxol Fast Blue-labeled myelin fibers in the cerebellum was evident at LPS9 (Figure 3D, *P* < 0.05).

We next searched for changes in neuronal density and morphology by examining brain regions such as the pons (mediator of cerebellar input and output) [57], the CA3 subregion of the hippocampus (responsible for short-term memory) [58], and the Purkinje cell layer that provides the output of all motor coordination in the cerebellar cortex [59] (representative images are showed in Figure 3A). The number of neurons was acutely decreased at LPS1 (*P* < 0.05), particularly in the pons where it was still evident at LPS9 (*P* < 0.05). The neuronal loss in both the hippocampus and cerebellum seen acutely at LPS1 was restored by LPS9 (Figure 3E). On the other hand, the marked soma reduction observed in neurons at LPS1 persisted until LPS9 in all three regions (Figure 3F, *P* < 0.01).

### LPS administration leads to a reduced myelination

**Figure 4:**
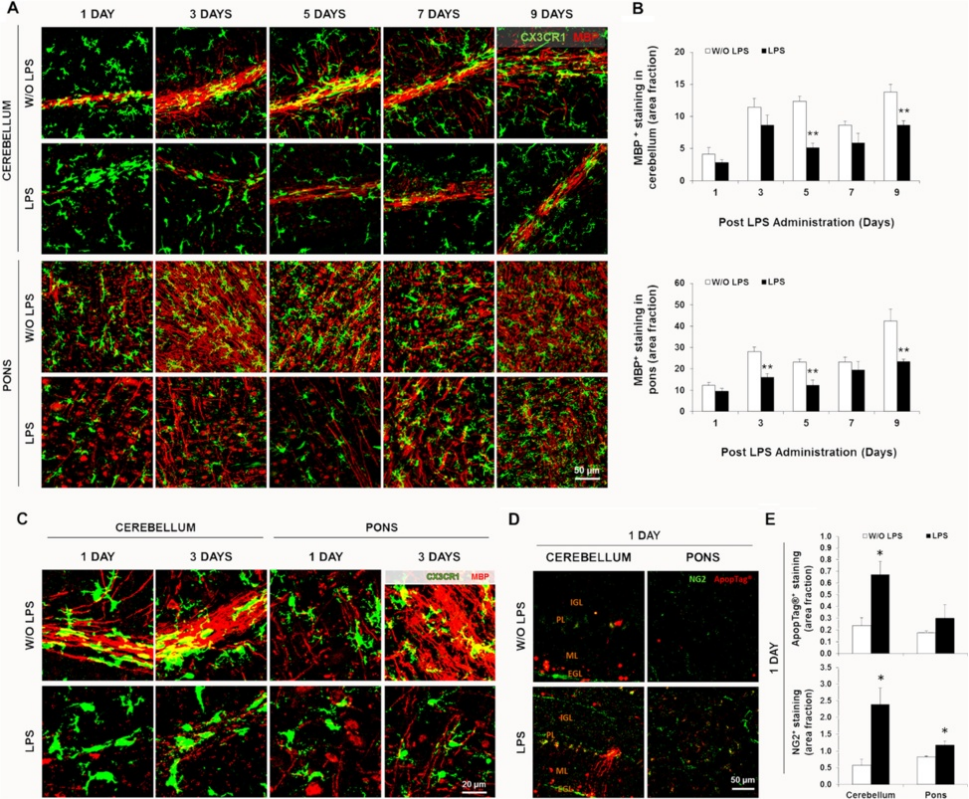
Lipopolysaccharide (LPS) administration disrupts myelination in the cerebellum and pons of neonatal mice. Brain cryosections from C57BL/6 CX3CR1^gfp/+^ mice at days 1/3/5/7/9 post-LPS administration were immunolabeled for myelin (myelin basic protein, MBP, red). Representative confocal images of the cerebellum and pons are displayed in **(A)**, where the overlapping of MBP and microglial marker CX3CR1 (green) is seen in yellow. **(B)** Area fraction per field of MBP staining was quantified per region using ImageJ software (NIH, USA). **(C)** To assess microglial phagocytosis, confocal images at days LPS1 and LPS3 were amplified (overlapping of MBP and CX3CR1 is visible in yellow). Sections of animals at LPS1 were immunolabeled for apoptosis (ApopTag, red) and oligodendrocytes precursor cells (NG2 cells, green). **(D)** Cerebellar layers are identified [external germinal layer (EGL), molecular layer (ML), Purkinje layer, internal granular layer (IGL)]. **(E)** Area fraction per field of ApopTag and of NG2 positive stainings were determined using ImageJ software (NIH, USA). *P < 0.05 and **P < 0.01 *vs*. without (W/O) LPS.

Given the significant impact on the myelin layer and the amount of neuronal damage evident in the pons and cerebellum, we further analyzed the effects of LPS on myelination in these two brain regions at days LPS1, LPS3, LPS5, LPS7, and LPS9 (representative images in Figure 4A). Reduced levels of MBP per unit area were observed at all time points, but the decrease was particularly evident at LPS5 (minimum values) and LPS9 in both brain regions when compared to controls (Figure 4B, *P* < 0.01). Considering the significant reduction of MBP at LPS5, we decided to investigate the contribution of CX3CR1^+^ microglia to this abnormality. We hypothesized that their phagocytotic activity might be associated with the delayed myelination. However, as shown in Figure 4C, this was not the case, as microglia did not contain cytoplasmic myelin signal at LPS1 or LPS3. To determine whether apoptosis of oligodendrocyte precursor cells (OPCs) could contribute to the decreased myelination, we stained tissues at LPS1 with ApopTag and anti-NG2 antibodies (expressed by OPCs) (Figure 4D). Increased apoptosis was observed in the cerebellum (*P* < 0.05) (particularly in the external germinal layer) as well as in the pons; however, no overlap of NG2^+^ labeling with ApopTag was noticed. Moreover, we observed an increased number of OPCs in the cerebellum (fourfold) and pons (1.4-fold) (Figure 4E, *P* < 0.05). These data suggest that there may be an increased proliferation of OPCs, or, alternatively, a delay in their maturation to myelinating cells resulting from neuroinflammation.

### Early neonatal LPS administration acutely decreases ATX levels while increasing other inflammatory biomarkers

**Figure 5:**
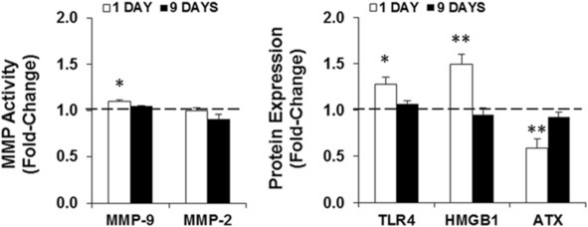
Lipopolysaccharide (LPS) challenge promotes differential expression of inflammatory biomarkers in the brain. Whole brain lysates of CD1 *wild-type* mice at 1 day post-lipopolysaccharide (LPS) administration were used to determine the activities of metalloproteinase(MMP)-9 and MMP-2 by gelatin zymography, alongside with the expression of toll-like receptor (TLR)-4, high-mobility group box 1 (HMGB1) and autotaxin (ATX) by western blot. Results are expressed as fold-change from animals without (W/O) LPS, and are mean ± SEM from at least four animals in each group. *P < 0.05 and **P < 0.01 *vs*. W/O LPS.

We next explored the neuroinflammatory reaction to systemic LPS injection by quantifying the expression of inflammatory biomarkers in whole brain tissue. The biomarkers MMP-9, MMP-2, TLR4, HMGB1, and ATX are known to be expressed by astrocytes, microglia, and/or BMECs during inflammatory conditions. We therefore evaluated if they were affected by the LPS administration and, if so, whether the effects were lasting (Figure 5). With the exception of MMP-2, all biomarkers were acutely modified by LPS administration. MMP-9 activity was modestly increased at LPS1 (*P* < 0.05), whereas TLR4 and HMGB1 expression levels were more elevated (*P* < 0.05 and *P* < 0.01, respectively). ATX expression, on the other hand, was significantly decreased (*P* < 0.01). The results indicate that the pro-inflammatory response observed 24 h after LPS administration was not sustained, as all biomarkers were restored to control levels at LPS9.

### Neonatal inflammation decreases the microglia transition from an amoeboid to ramified morphology

**Figure 6:**
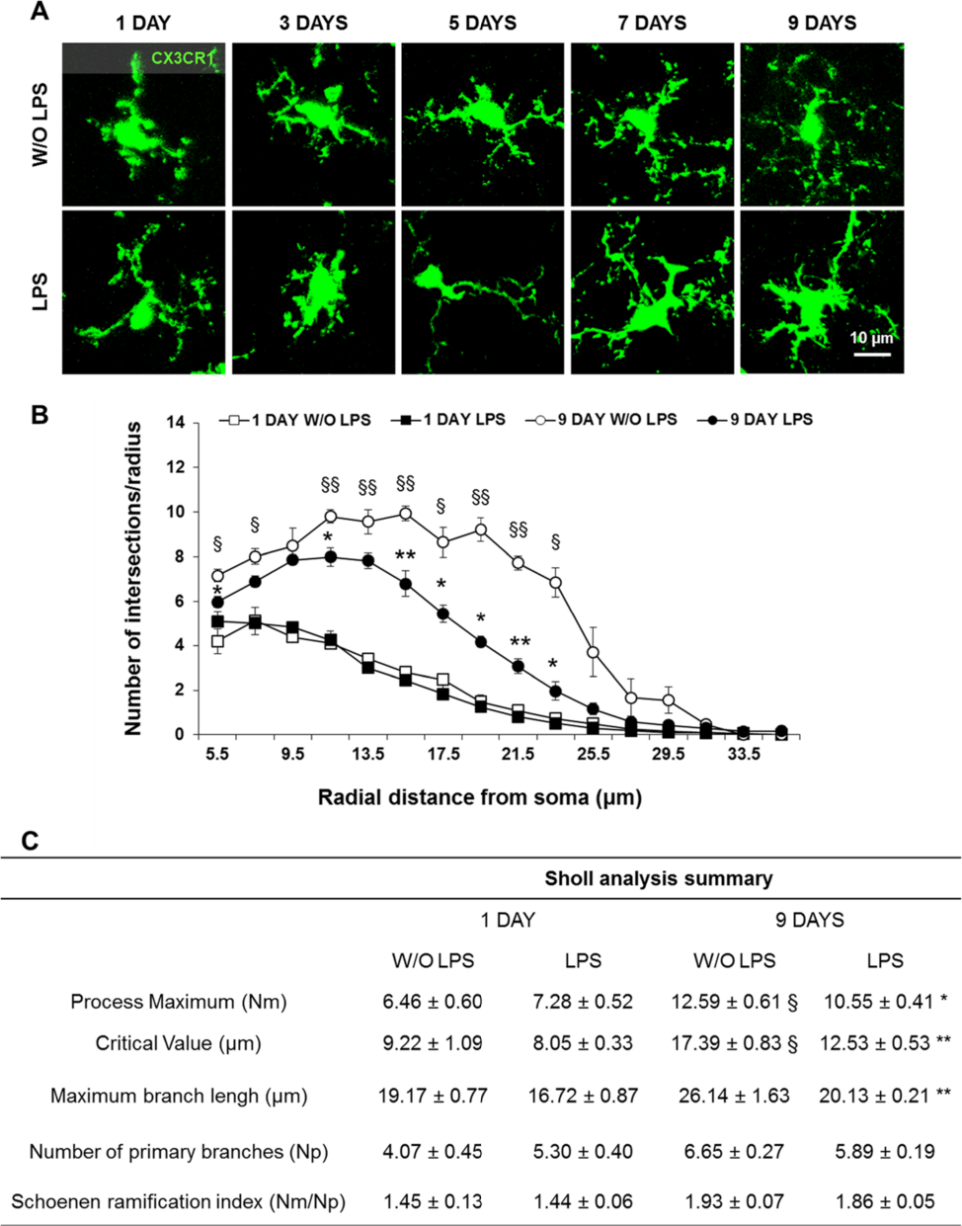
Morphological changes in microglia following lipopolysaccharide (LPS) administration. **(A)** Representative confocal images of CX3CR1^+^ cell morphology from brain cryosections of C57BL/6 CX3CR1^gfp/+^ mice at days 1/3/5/7/9 post-lipopolysaccharide (LPS) administration are shown. **(B**,**C)** Using ImageJ software (NIH, USA), we performed Scholl analysis (>40 cells per animal) at days 1 and 9 post-LPS administration. Results are mean ± SEM from at least four animals. *P < 0.05 and **P < 0.01 *vs*. without (W/O) LPS; ^§^ *P <* 0.05 and ^§§^ *P <* 0.01 *vs*. day 1 post-LPS.

The alterations in inflammatory biomarkers suggested that microglia might be involved in the response to peripheral LPS challenge. Therefore, we decided to examine microglial morphology following neonatal LPS administration in the pons - the brain region most affected by this endotoxin. Analysis of parenchymal CX3CR1^gfp/+^ cells in control mice revealed that microglia gradually changed morphology from an amoeboid phenotype at the end of the first week of life to a more ramified morphology by the second week (Figure 6A). This is supported by our quantitative data showing that the maximum number of microglia process interactions (process maximum, Nm) and the distance from the soma where these interactions occurred (critical value) increased over time in control mice (Figure 6B,C; *P* < 0.05 for Nm and *P* < 0.01 for critical value). However, these parameters were significantly decreased by LPS treatment. In fact, at LPS1 and LPS3, CX3CR1^+^ cells were still amoeboid. By LPS5, microglia showed elongated soma and few secondary processes, which progressed to further enlargement of the soma and a reduced number of short processes at LPS7 and LPS9 (Figure 6A). Quantitatively, at LPS9, the maximum radius at which a branch intersection occurred (maximum branch length) was significantly reduced (25%, *P* < 0.01). However, no alterations in the number of branches that originated from microglia soma (number of primary branches) or in the cell branching density (Schoenen ramification index) were noticed (Figure 6B,C).

### Decreased astrocytosis occurs after LPS5 and is inversely associated with microgliosis

**Figure 7:**
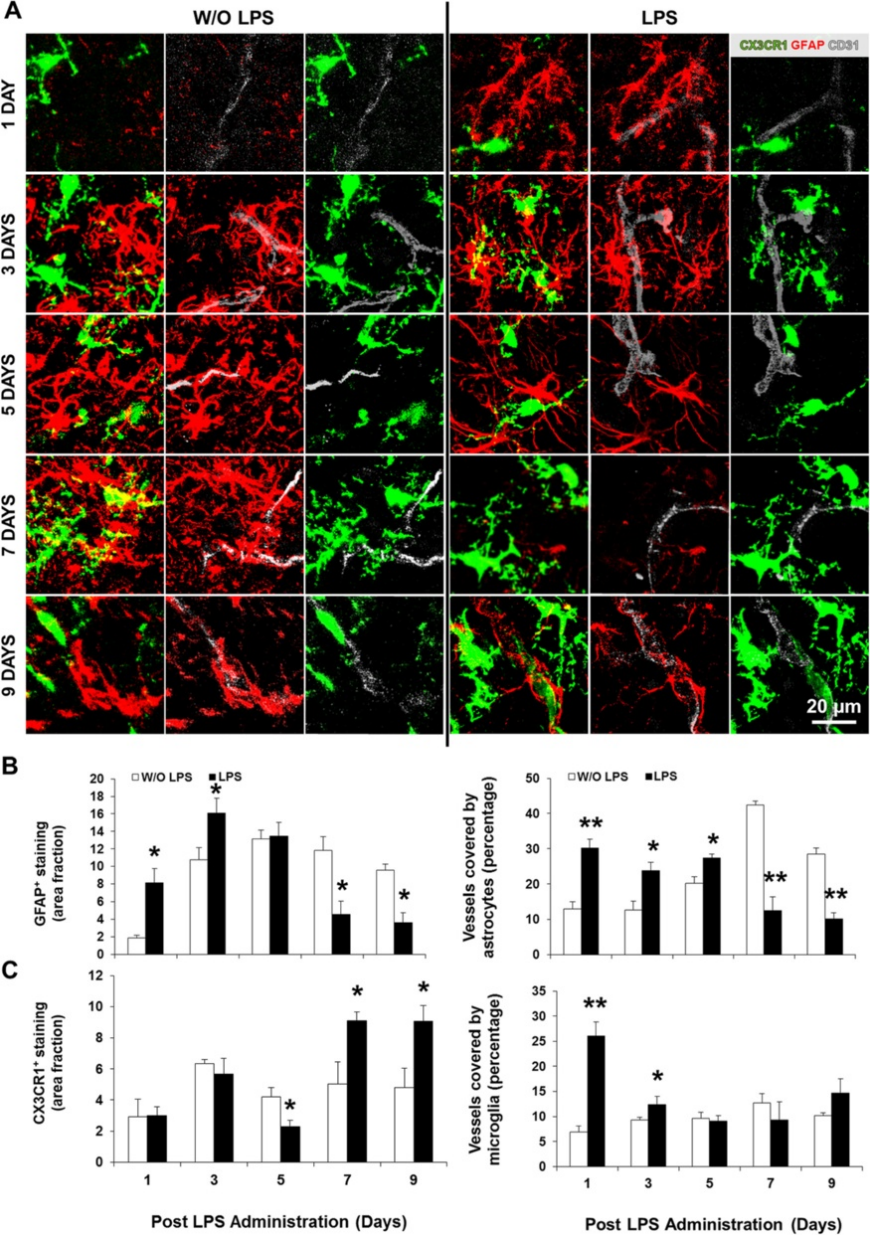
Early increased vessel coverage by glia is followed by delayed astrocytic loss and parenchymal microgliosis. Brain cryosections of C57BL/6 CX3CR1^gfp/+^ mice at days 1/3/5/7/9 post-lipopolysaccharide (LPS) administration were immunolabeled for astrocytes (glial fibrillary acidic protein, GFAP, red) along with vessel marker CD31 (cluster of differentiation 31, gray). Representative confocal images of glia-endothelium interactions in pons are displayed in **(A)**. **(B**,**C)** Area fraction per field of GFAP and CX3CR1 (green) positive staining, as well as their respective colocalization with the vessels were determined by ImageJ software (NIH, USA). Results are mean ± SEM from at least four animals. *P < 0.05 and **P < 0.01 *vs*. without (W/O) LPS.

To further evaluate the inflammatory reaction to LPS, we assessed glial responses (astrocytes and microglia, including their interaction with the vasculature) in the pons (Figure 7A). There was an acute reactive astrogliosis at LPS1 that extended until LPS3 in the parenchyma and to LPS5 around the microvessels (*P* < 0.05). By LPS7 and LPS9, the GFAP^+^ staining per unit in both locations was approximately half that of controls (Figure 7B, *P* < 0.05 in parenchyma and *P* < 0.01 at vasculature). The closest GFAP^+^-labeled area fraction to controls was at LPS5, when astrocytes showed long thin processes as seen in Figure 7A. Examination of microgliosis revealed that the area occupied by CX3CR1^+^ cells was markedly increased in association with the microvessels at LPS1 (*P* < 0.01) and LPS3 (*P* < 0.05) (Figure 7C). A transient decrease was observed in the pons parenchyma at LPS5, when microglia showed a dystrophic morphology (Figure 6A), followed by a remarkable increase at LPS7 and LPS9 (*P* < 0.05), but not around the microvessels (Figure 7C). Collectively, these results suggest that the kinetics of astrocytosis and microgliosis are inversely correlated in the parenchyma from LPS1 to LPS9.

### Increased density of CX3CR1^+^ cells around the vessels precedes the loss of GFAP^+^ cells in pons

**Figure 8:**
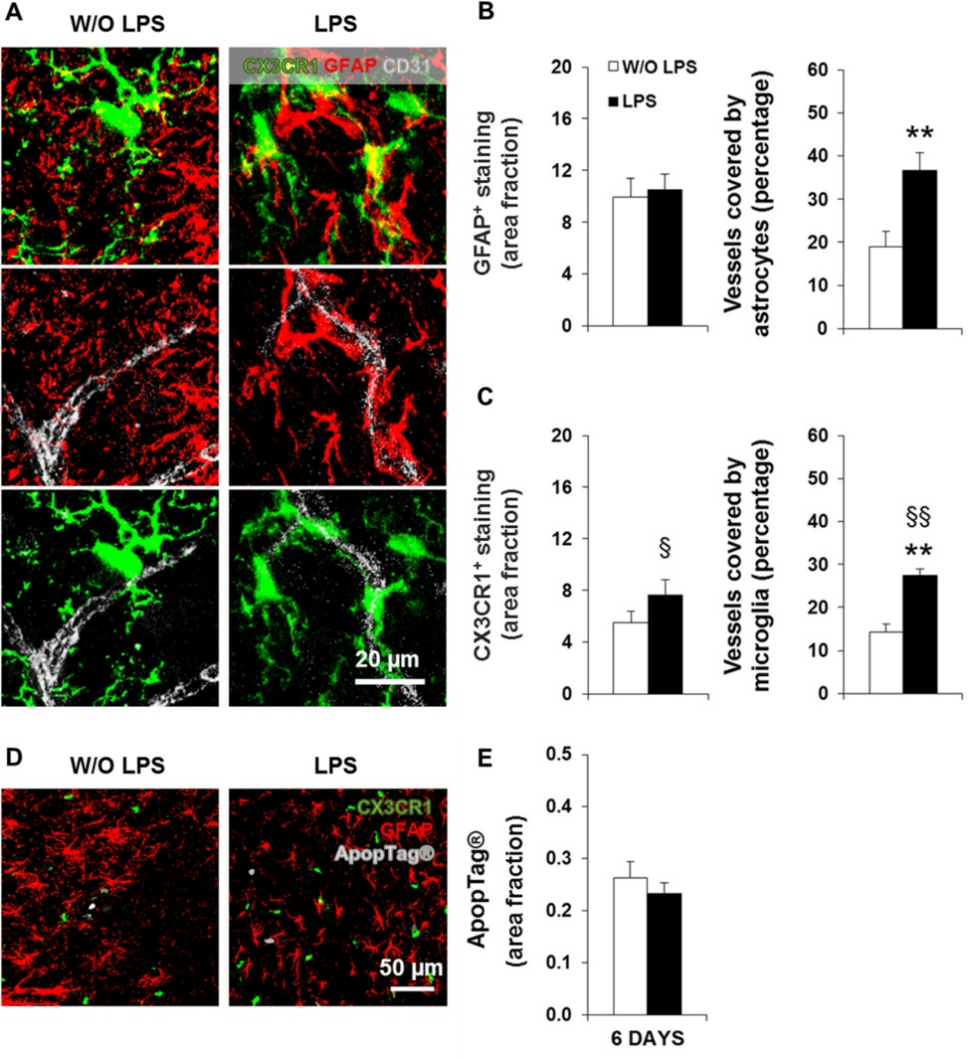
Astrocytic loss in neonatal inflammation is preceded by proliferation/migration of CX3CR1^+^ cells in the pons. Brain cryosections of C57BL/6 CX3CR1^gfp/+^ mice at day 6 post-lipopolysaccharide (LPS) administration were immunolabeled for astrocytes (glial fibrillary acidic protein, GFAP, red) and brain microvascular endothelial cells (cluster of differentiation, CD31, gray). Representative confocal images of each condition in the pons are depicted in **(A)**. **(B**,**C)** Area fraction per field of GFAP and CX3CR1 (green) positive staining, along with their respective colocalization with vessels, were determined using ImageJ software (NIH, USA). Representative confocal images of sections immunolabeled for astrocytes (GFAP, red) and for ApopTag (gray) are depicted in **(D)**. Quantification of ApopTag^+^ staining area fraction, using the abovementioned software, is shown in **(E)**. Results are mean ± SEM from at least four animals. **P < 0.01 *vs*. without (W/O) LPS; ^§^ *P <* 0.05 and ^§§^ *P <* 0.01 *vs*. day 5 post-LPS.

Our data pointed to a transition from astrocyte gain to loss between LPS5 and LPS7. In order to better understand this transition, we evaluated the glial-vasculature response at an intermediate time point - LPS6 (Figure 8A). At this time point, GFAP^+^ staining per unit in the parenchyma was similar to control and still higher around the vasculature (Figure 8B, *P* < 0.01) similar to LPS5 (Figure 7B). On the other hand, the parenchymal area occupied by CX3CR1^+^ cells at LPS6 had already increased to control levels (*P* < 0.05 compared to LPS5). Surprisingly, the area of microvasculature covered by these cells was significantly increased at this time point (Figure 8C, *P* < 0.01 compared to respective control and to LPS5), which was not visible either at LPS5 or at LPS7 (Figure 7C). These data suggest that CX3CR1^+^ cells migrate towards the microvasculature prior to the loss of perivascular GFAP^+^ cells. Lastly, we performed ApopTag staining at LPS6 to determine if the significant loss of GFAP^+^ staining at LPS7 was due to apoptosis. No difference in apoptotic cells was observed between the control and LPS-treated mice at this time point, nor did the ApopTag staining co-localize with microglia or astrocytes (Figure 8D,E). Thus, the reduction in GFAP^+^ staining at LPS7 likely results from a mechanism other than cell death.

## Discussion

Here, we demonstrate that repeated peripheral LPS administration in the first week of life, a condition that mimics septicemia in the premature human infants [7,60,61], alters normal mouse brain development over the following week. This leads to a delayed recovery of the LPS-induced neuronal atrophy as well as myelination deficits. These alterations reproduce the neuronal dysfunction, white-matter damage, and cerebral palsy associated with human perinatal brain injury following sepsis [62-66]. We also provide evidence that neonatal exposure to LPS causes a robust pro-inflammatory reaction in the CNS characterized by astrocyte and microglia activation followed by astrocyte loss. These structural and inflammatory changes may explain some of the CNS abnormalities observed in humans after neonatal sepsis [67,68].

Our study is novel in that it evaluates the effects of repetitive LPS injections (6 mg/kg of body weight) in mice from PND4 to PND6. Most previous studies were performed in adult or aged mice [20,44-69] and only one isolated administration of LPS was used [5,70] and at lower doses [71,72]. Lower LPS concentrations do not always elicit a septic response, and concentrations up to 25 mg/kg may be necessary depending on the mouse model [73]. In our study, we injected a high dose of LPS daily to induce a sustained septic state. This approach has been used by other investigators but not during the perinatal period [74,75]. Body weight, myelination, neuronal density, inflammatory biomarkers, and glial cell reactivity were evaluated for a week after the last injection to establish the impact of peripheral LPS challenge on the developing neonatal brain.

An acute loss in body weight following LPS administration is usually indicative of a sickness behavior in newborns [56], and a three injection regimen with 3 mg/kg in 6- to 8-week-old CD-1 mice resulted in significant weight loss, although the animals survived [75]. Loss of body weight is one of the consequences of sepsis [15], even in humans [16], and is a sign of illness in animal models [12-14]. We observed in our model that body weight decreased immediately following LPS injection. In a study by Du *et al.* [76], the effects of LPS in newborn mice were also studied, although the exposure period was longer (PND3 to PND11) and less LPS was administered per day (0.3 mg/kg). The animals examined at PND12 in this study were shown to have recovered their body weight. In our model, we observed a decrease in weight over the 7 days following the last LPS injection. Body weight returned to normal on day 9. Our findings are in line with a study showing weight loss in PND5 rats 24 h after LPS injection (2 mg/kg) [77].

We also observed an acute loss in brain weight following LPS injection. This is consistent with a study demonstrating that intrauterine administration of LPS (125 μg per dam) in mice at E15 reduces brain weight relative to controls, even at PND14 [78]. Again, this appears to be a dose-dependent effect given that intrauterine administration of lower LPS concentrations (80 μg/kg or approximately 3 μg per dam) did not induce brain weight loss in offspring at PND14 [79]. Nevertheless, this outcome is relevant to humans, as MRI scans have revealed that neonatal infection can be associated with changes in cerebral development, including a reduction in cerebral growth [66,67]. The overall loss of brain weight in our study may be linked in part to cerebellar hypoplasia. Several cerebellar regions were reduced in size following LPS administration, and our results suggest that this decrease might result from neuronal atrophy or decreased survival. Recent data demonstrate that LPS has stronger effect on cell survival, at least in the hippocampus, than on proliferation during inflammation in the neonatal mouse brain [80]. In addition, we observed that there was a decreased soma area and associated neuronal loss in all brain regions examined. The aforementioned study by Du *et al.* [76] also demonstrated a loss of NeuN^+^ neurons, although no change was observed in adult animals 24 h after LPS treatment (1 mg/kg) [81]. Thus, it is likely that the negative impact of LPS is more pronounced during neurogenesis and neuronal migration [7] because the developing brain is particularly sensitive to inflammation during this developmental period [80,82].

The negative impact of LPS also extended to the process of myelination when injected into neonatal mice. We observed that LPS caused a persistent decrease in MBP levels even at LPS9. Although most studies have only evaluated this parameter during the prenatal period [11,83,84], two recent reports documented hypomyelination following injection of low-dose LPS (0.05 mg/kg) at PND5 [85] and after repeated low-dose LPS (0.3 mg/kg) from PND3 to PND11 [76], which are consistent with our findings. In hypomyelinated mice, we did not observe evidence of phagocytic uptake of degraded myelin by CX3CR1^+^ myeloid cells but instead we saw an increased number of NG2^+^ cells. We postulate based on these findings that LPS slows or arrests oligodendrocyte differentiation instead of promoting myelin degradation. Additional studies are required to prove such theory.

Assessment of inflammatory biomarkers revealed that LPS triggers an increased expression of TLR4 and HMGB1, as well as enhanced MMP-9 activity, at LPS1. MMPs are gelatinases that have the capacity to remodel the extracellular matrix, promote cellular invasion, and induce various signaling pathways [86]. We previously showed that increased release of active MMP-9 and MMP-2 by BMECs occurred within 24 h of LPS exposure *in vitro* [29]. *In vivo*, we observed an elevated activation of MMP-9, but not of MMP-2, following LPS administration. Previous studies have shown that LPS-stimulated pericytes and microglia can lead to high levels of active MMP-9 [87], which has the potential to disrupt brain homeostasis, degrade the extracellular matrix, and ultimately weaken the BBB, giving rise to leakage [88]. Given that MMP-9 can open the BBB [89] and LPS induces its expression through the TLR4/NF-κB pathway [90], we investigated whether this receptor was upregulated after the LPS challenge. We observed increased expression of TLR4 at LPS1 but not at LPS9. TLRs are expressed in immune cells, microglia, as well as in BMECs and respond to microbial infections. TLR4 is the receptor responsible for the initial inflammatory response to LPS and is usually elevated within hours of exposure [91]. Although few studies have focused on TLR4 expression in newborn mice, a recent one reported that TLR4 was elevated 24 h after exposure to 1 mg/kg of LPS in 6-week-old mice [81], what is in agreement with our data.

Interestingly, it was demonstrated that HMGB1 triggers MMP-9 upregulation in neurons and astrocytes predominantly *via* TLR [92,93]. HMGB1 is a nuclear protein and an alarmin that is secreted by immune cells and endothelial cells, as well as neurons, microglia, and astrocytes, in response to an inflammatory stimulus [94]. HMGB1 was acutely elevated in the brain homogenates at LPS1, which would potentiate TLR4 signaling. Previous studies have demonstrated that this biomarker is markedly increased during neonatal inflammation [95,96]. HMGB1 was shown to contribute to ‘sickness’ behavior and likely causes decreased food intake [97]. This could explain the body weight reduction observed in our study. In contrast to the increase in MMP-9, TLR4, and HMGB1 levels, a decrease in ATX expression was noticed at LPS1, despite studies showing that its expression is normally elevated in inflammatory diseases [98]. ATX has been associated with an anti-inflammatory and defensive role, at least in microglia [38,99]. Further studies are required to determine the exact role played by ATX in the developing neonatal brain following a peripheral LPS challenge.

Given the increased expression of inflammatory biomarkers during neonatal sepsis, we decided to evaluate glial reactivity and their interactions with the brain microvasculature. Both astrocytes and microglia became reactive and were associated with increased coverage of blood vessels early after LPS administration. This response waned over time, with astrocytes eventually showing reduced vascular interactions. A sustained glial response to LPS was recently described in the hippocampus and brainstem of adult mice following induction of sepsis [100]. In addition, it has been shown that microglia and astrocytes proliferate in response to LPS [81,101] and *E. coli* [102]. Our results are in agreement with the findings of Gómez-Nicola *et al.* [103], who showed that LPS administration alone can induce reactivity of both microglia and astrocytes. It is conceivable that the neuronal atrophy and delayed myelination observed in our study is in fact linked to the LPS-induced glial response. Astrocytes and microglia are essential for the formation, trimming, and function of developing synapses [104,105], as well as for CNS myelination by promoting OPC migration, proliferation, and differentiation [106]. Studies have reported that the loss or dysfunction of astrocytes can lead to demyelination or inhibited oligodendrocyte maturation [107,108]. Others have also shown that disruption of microglia-mediated synaptic pruning contributes to neurodevelopmental disorders [109] and produces long-lasting defects in oligodendrocyte maturation and myelination [110,111].

The divergent early and late astrocytic responses to LPS administration may be linked to the concept of tertiary brain damage [112], especially given the role that astrocytes play in brain homeostasis and BBB maintenance. Sherwin *et al.* [113] showed that LPS binds to microglia and astrocytes during LPS-induced neonatal neuroinflammation, and the response was particularly intense on astrocytes surrounding blood vessels. LPS was shown to increase BBB resistance by inducing a protective response in endothelial cells and astrocytes [114]. Therefore, the initial increased astrocytic coverage of microvessels here reported is in line with a protective neuroinflammatory reaction. With time, the upregulation of the chemoattractant CX3CL1 by astrocytes in response to inflammatory mediators [115] may attract microglia, which is supported by the increased CX3CR1^+^ staining and re-distribution we observed. Interestingly, the reduced association of GFAP^+^ cells with microvessels over time coincided with the morphological transformation of microglia into a bushy, activated state. This is consistent with studies showing that reduced astrocytosis may follow a concomitant increase in microgliosis after a neuroinflammatory stimulus [116,117].

## Conclusion

Our data demonstrate that systemic inflammation profoundly alters several anatomical and inflammatory aspects of the brain when experienced during the early neonatal period. Cerebellar hypoplasia and neuronal atrophy coincided with reduced myelination, delayed differentiation of microglia from an amoeboid into the ramified state, and enhanced glial coverage of cerebral blood vessels. This was followed by decreased astrocytosis and increased microgliosis. Our results not only expand upon previous literature but are also the first to document the ongoing progression of neuroinflammatory changes that occur in the week that follows repeated LPS administration. Future studies on the neurodevelopmental outcome and glial response to a second LPS stimulus in young adult mice will help elucidate tertiary mechanisms of damage and whether or not the brain becomes sensitized to further injury. These studies should hopefully aid in the development of therapies that promote the functional recovery of microglia and astrocytes in the inflamed brain following neonatal sepsis.

## Abbreviations

ATX: autotaxin
BBB: blood-brain barrier
BMECs: brain microvascular endothelial cells
CD31: cluster of differentiation 31
CNS: central nervous system
CX3CR1: fractalkine receptor
GFAP: glial fibrillary acid protein
GFP: green fluorescent protein
HMGB1: high-mobility group box 1
LPS: lipopolysaccharide
MBP: myelin basic protein
MMP: matrix metalloproteinase
PND: postnatal day
TLR4: toll-like receptor 4.
